# Condyloma acuminata: An evaluation of the immune response at cellular and molecular levels

**DOI:** 10.1371/journal.pone.0284296

**Published:** 2023-04-13

**Authors:** Bruna Stuqui, Paola Jocelan Scarin Provazzi, Maria Leticia Duarte Lima, Ágata Silva Cabral, Ellen Cristina Rivas Leonel, Natalia Maria Candido, Sebastião Roberto Taboga, Márcia Guimarães da Silva, Flávio de Oliveira Lima, Patrícia Pereira dos Santos Melli, Silvana Maria Quintana, Marilia de Freitas Calmon, Paula Rahal

**Affiliations:** 1 Department of Biology, São Paulo State University, São José do Rio Preto, São Paulo, Brazil; 2 Department of Histology, Embryology and Cell Biology, Federal University of Goiás, Goiânia, Goiás, Brazil; 3 Department of Pathology, São Paulo State University, Botucatu, São Paulo, Brazil; 4 Clinical Hospital of Ribeirāo Preto, University of São Paulo, Ribeirão Preto, São Paulo, Brazil; 5 Department of Gynecology and Obstetrics, Ribeirāo Preto Medical School, University of São Paulo, Ribeirão Preto, São Paulo, Brazil; University of Hong Kong, HONG KONG

## Abstract

Condyloma acuminata (CA) is a benign proliferative disease mainly affecting in non-keratinized epithelia. Most cases of CA are caused by low-risk human papillomavirus (HPV), mainly HPV 6 and 11. The aim of the current study was to highlight the candidate genes and pathways associated with immune alterations in individuals who did not spontaneously eliminate the virus and, thus, develop genital warts. Paraffin-embedded condyloma samples (n = 56) were analyzed by immunohistochemistry using antibodies against CD1a, FOXP3, CD3, CD4, CD8, and IFN-γ. The immunomarkers were chosen based on the evaluation of the innate and adaptive immune pathways using qPCR analysis of 92 immune-related genes, applying a TaqMan Array Immune Response assay in HPV 6 or HPV 11 positive samples (n = 27). Gene expression analysis revealed 31 differentially expressed genes in CA lesions. Gene expression validation revealed upregulation of *GZMB*, *IFNG*, *IL12B*, and *IL8* and downregulation of *NFATC4* and *IL7* in CA samples. Immunohistochemical analysis showed increased FOXP3, IFN-γ, CD1a, and CD4 expression in CA than in the control tissue samples. In contrast, CD3 and CD8 expression was decreased in CA lesion samples. Increased levels of pro-inflammatory cytokines in HPV-positive patients compared with HPV-negative patients seem to reflect the elevated immunogenicity of HPV-positive CA lesions. Host defense against HPV begins during the early stages of the innate immune response and is followed by activation of T lymphocytes, which are mainly represented by CD4+ and regulatory T cells. The low CD8+ T cell count in CA may contribute to this recurrent behavior. Additional studies are needed to elucidate the mechanism of host defense against HPV infection in CA.

## Introduction

Human papillomavirus (HPV) infection is related to several benign and malignant diseases [1–4]. The pathology in HPV infections depends on the virus type, host immune response, and local environmental factors [5].

Condylomata acuminata, or anogenital warts, is a sexually transmitted infection (STI) caused by HPV, most often by low-risk HPV types 6 and 11; however, coinfections with high-risk HPV types can also be observed [6].

Anogenital warts are benign proliferative diseases that manifest as visible lesions, such as single or multiple papules on the vulva, vagina, cervix, penis, scrotum, perineum, and perianal area [7].

Although condyloma acuminata is a benign disease, condylomas cause significant psychosocial distress and anxiety [5]. The treatment involves tissue destruction and use of topical immune-modulating, antimitotic, antiviral, and anticarcinogenic medications. Surgical excision or destruction by freezing, diathermy, CO_2_ laser vaporization, and interferon injections are also frequently used [7]. However, condyloma shows a high incidence of recurrence (13–65%), thus requiring multiple treatments [5, 8].

In a generic viral skin infection, keratinocytes, considered as sentinels in the innate immune response, express pattern recognition receptors (PRR) that recognize viral DNA or RNA [9]. These receptors, known as Toll-like receptors (TLRs), are expressed on the cell surface or in endosomes, and recognize pathogen-associated molecular patterns (PAMPs). TLRs present in endosomes promote the production of type I interferon (IFN) and pro-inflammatory cytokines via IFN regulatory factors (IRFs) and nuclear factor kappa-light-chain-enhancer of activated B cells (NF-κB), thus activating different immune pathways [10].

The immune response to HPV is based on recognition by epidermal dendritic cells, represented by Langerhans cells (LCs) [11]. Langerhans cells play an essential role in recruiting innate and adaptive immune cells to eliminate pathogens [12]. During maturation, after viral recognition, these dendritic cells begin expressing surface molecules, such as CD83 and MHC (class I and II), which are required for antigen presentation, along with costimulatory molecules, CD80 and CD86, and release pro inflammatory cytokines. These cells become responsive to chemokines CCL19 and CCL21 via the CCR7 receptor, allowing their migration to the lymph nodes. After migration, Langerhans cells activate immature T cells that target the infected tissue for initiating an adaptive immune response [12, 13].

T cells, helper T (Th) lymphocytes, and cytotoxic T lymphocytes are responsible for regulating the adaptive immune response in cooperation with antigen-presenting cells. The release of different cytokines during infection regulates the synthesis and action of other cytokines. The Th1 immune response is responsible for the release of immunostimulatory cytokines, such as interferon gamma (IFNG), TNF alpha, interleukin 2, and interleukin 12, which mainly induce cell-mediated immunity. Th2-type cytokines (IL 4, IL 5, IL 6, IL 8, and IL 10) predominantly induce humoral immune responses and inhibit cell-mediated responses) [14–17].

Previous studies have demonstrated that the occurrence, remission, relapse, and cancerization of condyloma acuminata are associated with immune disorders in patients [18, 19]. Even in patients who are not immunosuppressed, benign HPV-mediated lesions are difficult to eradicate; therefore, these lesions can exhibit local immunosuppression in healthy individuals [19].

In the present study, we performed expression profiling of 92 immune-related genes in condyloma acuminata biopsies. We also evaluated the population of immune cells present in the condyloma microenvironment, aiming to highlight candidate genes and pathways associated with immune changes in individuals that did not spontaneously eliminate the virus and thus developed genital warts.

## Material and methods

### Ethics statement and samples

All procedures involving human participants in this study were in accordance with the ethical standards of the institutional and/or national research committee and with the 1964 Helsinki declaration and its later amendments or comparable ethical standards. Written informed consent was obtained from all participants included in the study. The study was approved by the Research Ethics Committee of the Institute of Biosciences, Humanities and Exact Sciences of São Paulo State University, city of São José do Rio Preto, Brazil (license number 37830514.0.0000.5466). All the patients agreed to participate in the study.

Gene expression analyses were carried out using 27 fresh condyloma acuminata (CA) biopsies with the respective adjacent normal tissues, obtained by surgical excision from female patients who visited the Clinical Hospital of the University of São Paulo Medical School, Ribeirão Preto, Brazil. For these analyses, only HIV-negative patients were considered, and the biopsies were subjected to histopathological analysis by an experienced pathologist who diagnosed CA.

Seventy paraffin-embedded samples were used for immune cell quantification using immunohistochemistry. These included 56 biopsies of CA and 14 normal vulvovaginal tissue samples obtained from HPV-negative patients undergoing myomectomy, which were used as control samples. These samples were obtained from the Medical School (FMB) of São Paulo State University (, São Paulo Brazil), and CA was histopathologically diagnosed by an experienced pathologist.

### Nested polymerase chain reaction (NESTED-PCR) and HPV genotyping

The methodology for Total DNA and RNA isolation is described in the supplementary material.

Polymerase chain reaction (PCR) was used to amplify HPV DNA in 27 condyloma samples. The oligonucleotides PGMY09 (5-CGT CCM ARR GGA WAC TGATC- 3) and PGMY11 (5-GCM CAG GGW CAT AAY AAT GG-3) were used as degenerate primers to detect viral DNA. The amplification products were used for nested PCR with the primers GP5+ (5′-TTTGTTACTGTGGTAGATACTAC-3′) and GP6+ (5′-CTTATACTAAATGTCAAATAAAAA-3′), which amplify a sequence of 150 bp internal to the fragment produced by the PGMY09/PGMy11 pair of oligonucleotides [20]. The amplifications were performed in a final volume of 25.0 μl containing 2.5 U of Taq DNA Polymerase (Sinapse Inc., Florida, USA, Catalog number: P1011-1), 2.5 μl of 10X PCR Buffer, 5.6 μM MgCl_2_, 0.2 mM dNTPs, 0.4 μM of each primer, 500 ng of DNA, and nuclease-free water. Thermocycling conditions included an initial denaturation step at 95°C for 9 min, followed by 40 cycles at 95°C for 1 min, 55°C for 1 min, 72°C for 2 min, and a final extension at 72°C for 5 min. For nested PCR, the 25.0 μL amplification reaction comprised 2.5 U of Taq DNA Polymerase (Sinapse Inc., Florida, USA, Catalog number: P1011-1), 2.5 μL of 10X PCR Buffer, 7.6 μM magnesium chloride (MgCl_2_), 0.064 mM deoxynucleotides (dNTPs), 0.48 μM of each primer, 5.0 μL of the product from the PGMy09/PGMy11 reaction, and nuclease free water. An initial denaturation step was conducted at 95°C for 9 min, followed by 40 cycles at 94°C for 30 s, 45°C for 30 s, and 72°C for 30 s, and a final extension at 72°C for 8 min. Positive (Caski cell line) and negative controls (Nuclease-free water) were used in the PCR.

The amplification products were electrophoresed on a 1% agarose gel. Sanger sequencing was performed to assess the HPV genotype [21]. The reactions were processed in 10.0 μL containing:2.0 μL of Big Dye Terminator (Applied Biosystems/Life Technologies, Foster City, CA, USA, Catalog number:4337457), 2.0 μL of Sequencing Buffer (5X), 1.0 μL of 10 pmol GP5+ and GP6+ oligonucleotides, and 5.0 μL of sample. The samples were subjected to initial denaturation at 95°C for 10 min, followed by 25 cycles of 20 s at 95°C, 20 s for oligonucleotide annealing at 50°C, and 1 min at 60°C for chain extension. A sequence of 34 bases downstream of the GP5+-binding site was used for accurate genotyping of the HPV type [22].

The obtained sequences were assessed for quality using PHRED/PHRAP/CONSED, available online at http://asparagin.cenargen.embrapa.br/phph/, and then aligned and checked for similarity with the sequences deposited in GenBank using BLAST–(Basic Local Alignment System) [23]. All sequences were edited using BioEdit (Biological Sequence Alignment Editor) [24].

### Real-time quantitative PCR—RT-qPCR

Gene expression in CA samples and adjacent tissue samples without lesions was compared. Lesion margins were used to mitigate the bias in gene expression analysis between CA and normal tissues.

To analyze immune-related gene expression and the biological component of variation, 27 fresh CA samples were synthetized and combined in three cDNA pools; the first, second, and third pools were prepared with six samples of HPV 6 condylomas, six samples of HPV 11 condylomas, and six HPV-negative sample margins, respectively. Therefore, each pool was tested as a single sample (one sample HPV 6, one sample HPV 11, and one negative sample) in TaqMan array RT-qPCR.

Twenty-seven cDNA samples, including those used in the PCR array, were used to validate the results of the PCR array using RT-qPCR.

cDNA samples were prepared from 1 μg of total RNA using the High-Capacity cDNA Reverse Transcription kit (Applied Biosystems, Carlsbad, California, USA, catalog number:4368814). The reactions were prepared in a final volume of 20 μL, containing 1x Buffer, 1x dNTP Mix, 1x Random Primers, Oligo(dT)_12-18_ (Invitrogen, Carlsbad, California, USA, Catalog number:18418012) (0.25 μM), RNaseOut (Recombinant Ribonuclease Inhibitor) (Invitrogen, Carlsbad, California, USA, Catalog number:10777019) (5 mM), and MultiScribe Reverse Transcriptase (2,5 U). The tubes were maintained at 25°C for 10 min followed by incubation at 37°C for 120 min.

RT-qPCR was carried out using a TaqMan^™^ Array, Human Immune, Fast 96-well plate (Applied Biosystems, Carlsbad, California, USA, Catalog number:4418718), representing 92 immune-related genes and four reference genes (S1 Table), according to the manufacturer’s instructions. Briefly, 20 ng of each cDNA pool was mixed with 2x Master mix in a final volume of 10 μL. Thermal cycling was conducted in two initial incubations at 50°C for 2 min and 95°C for 20 s, followed by 40 cycles at 95°C for 1 s and 60°C for 20 s.

For TaqMan array validation, 20 ng of the 27 condyloma cDNA samples were analyzed individually using TaqMan Fast Advanced Master Mix (Applied Biosystems, Carlsbad, California, USA, Catalog number:4444963) and TaqMan Gene Expression Assays (Applied Biosystems, Carlsbad, California, USA, Catalog number:4331182) (S2 Table), according to the manufacturer’s protocol. The HPV-negative cDNA pool was used as a reference sample, and *HPRT1* was the endogenous gene.

RT-qPCR was performed on a QuantStudio 12 K Flex Real-Time PCR System (Applied Biosystems, Carlsbad, California, USA, Catalog number 4471087). All samples were tested in triplicate. The relative expression of each specific gene was calculated using the ΔΔCq method [25]; a cutoff of > 2-fold change was considered significant.

### Tissue microarrays

Seventy paraffin-embedded samples were arranged in two tissue microarrays (TMAs): one corresponding to case-patients with lesions, and the other from control-patients. The case-patients TMA contained 56 biopsies of CA. The control-patients TMA contained 14 vulvovaginal tissue samples obtained from healthy patients undergoing myomectomy.

### Immunohistochemical assays

Immunohistochemistry was performed to detect six immunomarkers (S3 Table). Slides were deparaffinized in xylol and rehydrated in a series of decreasing ethanol concentrations. Antigen recovery was performed for 45 min at 96°C in a citrate buffer (pH 6.0). Next, the slides were washed (TBS-T) thrice for 5 min each and incubated with 0.1% H_2_O_2_ ready for use solution (ab80437 Abcam, UK) for 10 min. Protein blocking solution was applied for 10 min, and after washing in TBS-T, the slides were incubated overnight at 4°C with primary antibodies (diluted in 1% albumin). Slides for detecting FOXP3, CD1a, CD4, and CD8 (Abcam, UK) were incubated with HRP polymer (ab210059, Abcam, UK) for 30 min at 25°C, and those for CD3 and IFN-γ (Abcam, UK were incubated with a secondary HRP-conjugated rabbit antibody (ab80437, Abcam, UK) for the same time (specific targets and dilutions of the primary antibodies are shown in S3 Table). For rabbit-raised antibodies, a biotin-free immunoenzymatic detection kit was used (Expose Rabbit Specific HRP/DAB Detection Kit, ab80437 Abcam, UK), whereas for mouse-raised antibodies, a double-stain IHC kit (M&R on human tissue, DAB & AP/Red, ab210059, Abcam, UK) was used. The slides were stained with the chromogenic substrate DAB (3,3’-diaminobenzidine tetrahydrochloride) (ab210059, Abcam, UK) and counterstained with hematoxylin.

### Immunostaining quantification

Each slide was digitized at 20x magnification using a BX61VS camera (Olympus, Japan) coupled with an Olympus VS120 Virtual Microscope Slide Scanning System (VS120-S5). For quantifying FOXP3 and IFN-γ proteins, which presented diffuse cytoplasmic staining in the epithelium, three random photomicrograph fields of each TMA sample were captured using OLYMPUS OlyVIA 2.9 software (Olympus Corporation, Japan) at 40x magnification. Immunostaining was quantified by imaging analysis using Image-Pro Plus software (version 6, Bethesda MD, USA), with the M130 multipoint system proposed by Weibel, [26] as previously described by Antoniassi et al. [27]. The epithelial and stromal regions of each tissue were evaluated, the expression of FOXP3 and IFN-γ was quantified, and the incidence of positive immunostaining was presented as a percentage.

For the cellular and punctual staining markers CD1a, CD3, CD4, and CD8, the number of positive cells per area was noted along with the values expressed in cells per square millimeter of tissue (cell/mm²). Quantification was also performed on the digitized slides. For each sample, positive brown-stained cells were counted individually using OLYMPUS OlyVIA 2.9 software (Olympus Corporation, Japan). The total areas of the TMA samples were measured.

### Statistical analysis

Data were subjected to the Kolmogorov-Smirnov normality test and found to be normally distributed; all groups (case-patients and control-patients) were compared using Analysis of Variance (ANOVA) followed by Tukey’s multiple comparison test. All values are expressed as the mean ± standard error using GraphPad Prism 8 (GraphPad Software Inc., San Diego, CA, USA). Any *p-value* ≤ 0.05 was considered statistically significant.

## Results

### HPV genotyping and immune-related gene expression in condyloma samples

Among the 27 CA HPV-positive samples evaluated, 29.6% showed the HPV 11 genotype and 70.4% showed the HPV 6 genotype, which was the most prevalent (S4 Table).

**Fig 1:**
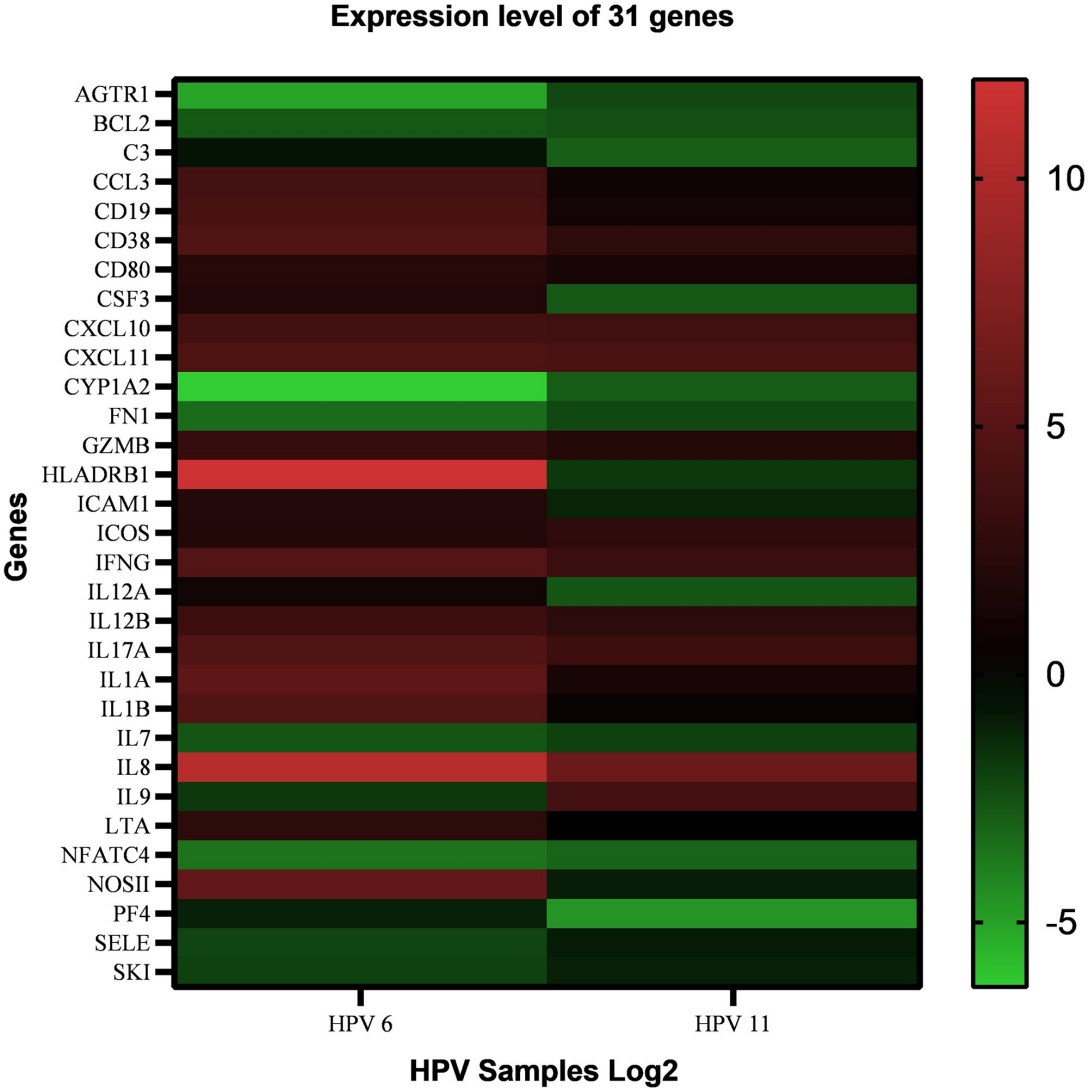
Heatmap of 31 differentially expressed genes in condyloma acuminata samples. A numerical version of Fig 1 is presented in S6 Table.

Array analysis of 92 immune-related genes (S5 Table) revealed 31 differentially expressed genes in condyloma samples compared to those in control tissues, considering a significance threshold of 2-fold or -2-fold expression (Log_2_) (Fig 1 and S6 Table). Eight of these genes, *AGTR1*, *BCL2*, *CYP1A2*, *FN1*, *IL7*, *NFATC4*, *SELE*, *SKI*, were downregulated in the pool of HPV 6 condyloma samples, while nine, *AGTR1*, *BCL2*, *C3*, *CSF3*, *CYP1A2*, *FN1*, *IL12A*, *NFATC4*, *PF4*, were downregulated in the pool of HPV 11 condyloma samples (S6 Table). Five genes (*AGTR1*, *BCL2*, *CYP1A2*, *FN1*, *NFATC4*) were downregulated in the pools of both HPV 6 and HPV 11 condyloma samples. Seventeen genes (*CCL3*, *CD19*, *CD38*, *CD80*, *CXCL10*, *CXCL11*, *GZMB*, *HLADRB1*, *ICAM1*, *IFNG*, *IL12B*, *IL17A*, *IL1A*, *IL1B*, *IL8*, *LTA*, *NOS2*) presented increased expression in the pool of HPV 6 condyloma samples and ten genes (*CD38*, *CXCL10*, *CXCL11*, *GZMB*, *ICOS*, *IFNG*, *IL12B*, *IL17A*, *IL8*, and *IL9*) showed increased expression in the pool of HPV 11 condyloma samples. Eight genes were upregulated in the pools of both HPV condyloma samples (*CD38*, *CXCL10*, *CXCL11*, *GZMB*, *IFNG*, *IL12B*, *IL17A*, *IL8*) (S6 Table).

**Fig 2:**
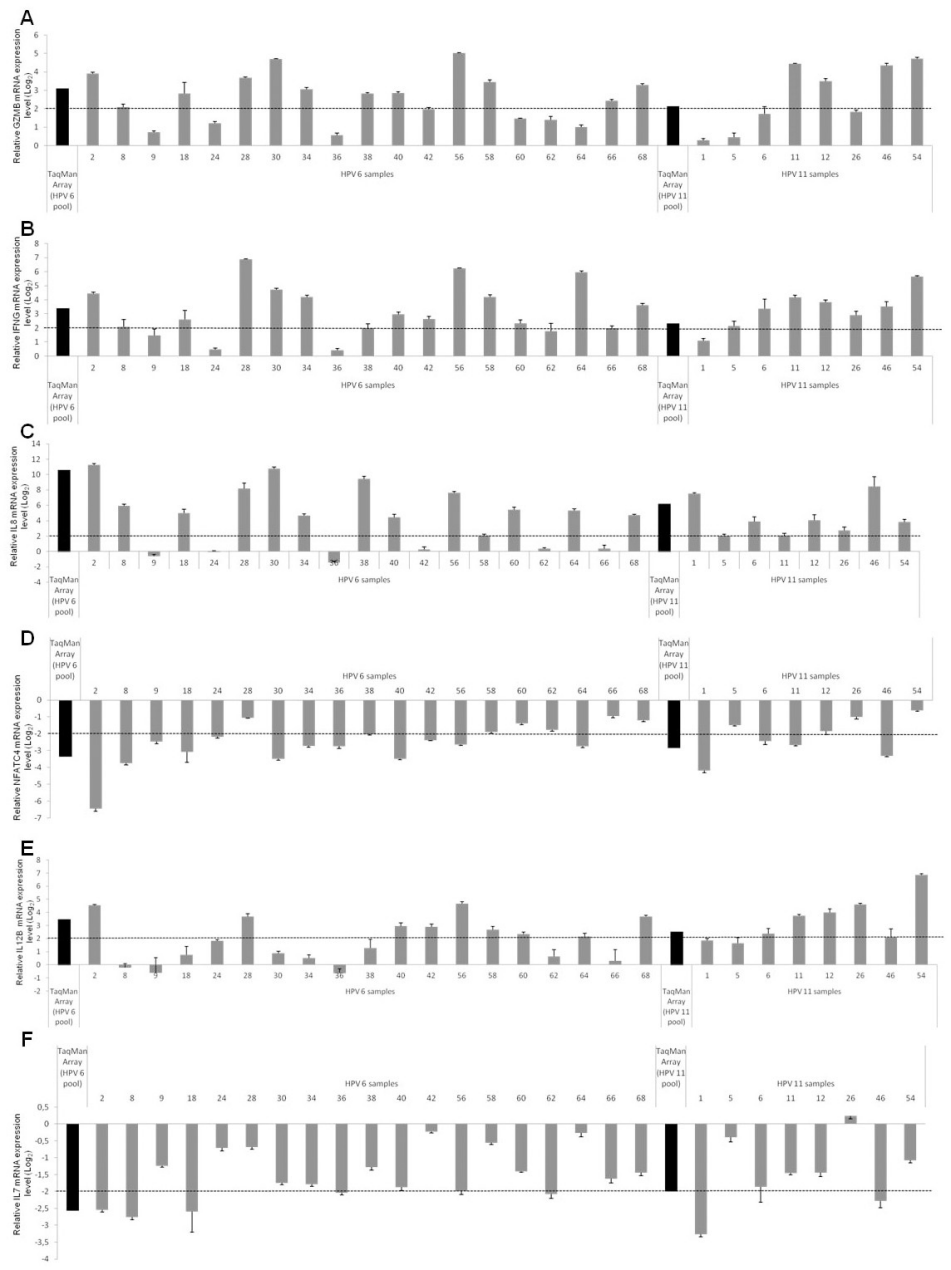
A–F: Validation using RT-qPCR of immune-related genes that were differentially expressed in condyloma acuminata. The expression of *GZMB* (A), *IFNG* (B), *IL8* (C), *NFATC4* (D), *IL12B* (E), and *IL7* (F) in condyloma samples (HPV6 positive and HPV 11 positive) compared to that in the control (HPV negative samples) is shown as fold change (log2) relative to expression.

Six of these genes, *GZMB*, *IFNG*, *NFATC4*, *IL12B*, *IL7*, and *IL8*, were selected for qPCR validation using 27 condyloma samples (S2 Table). The choice of genes was based on a literature review, according to their roles in innate and adaptive immunity [28–33]. *GZMB* was upregulated in 63% of the samples (17/27), similar to the TaqMan Array result (Fig 2A). Similarly, *IFNG* was upregulated in 81.5% (22/27) of the samples (Fig 2B), and *IL8* was upregulated in 78% (21/27) of the samples (Fig 2C). *NFATC4* was downregulated in 63% of the samples (17/27) (Fig 2D). *IL12B* was upregulated in 55.5% of the biopsies (15/27) (Fig 2E), whereas *IL7* was downregulated in only 30% (8/27) of the samples (Fig 2F).

### Incidence of immune cells in the lesion microenvironment

**Fig 3:**
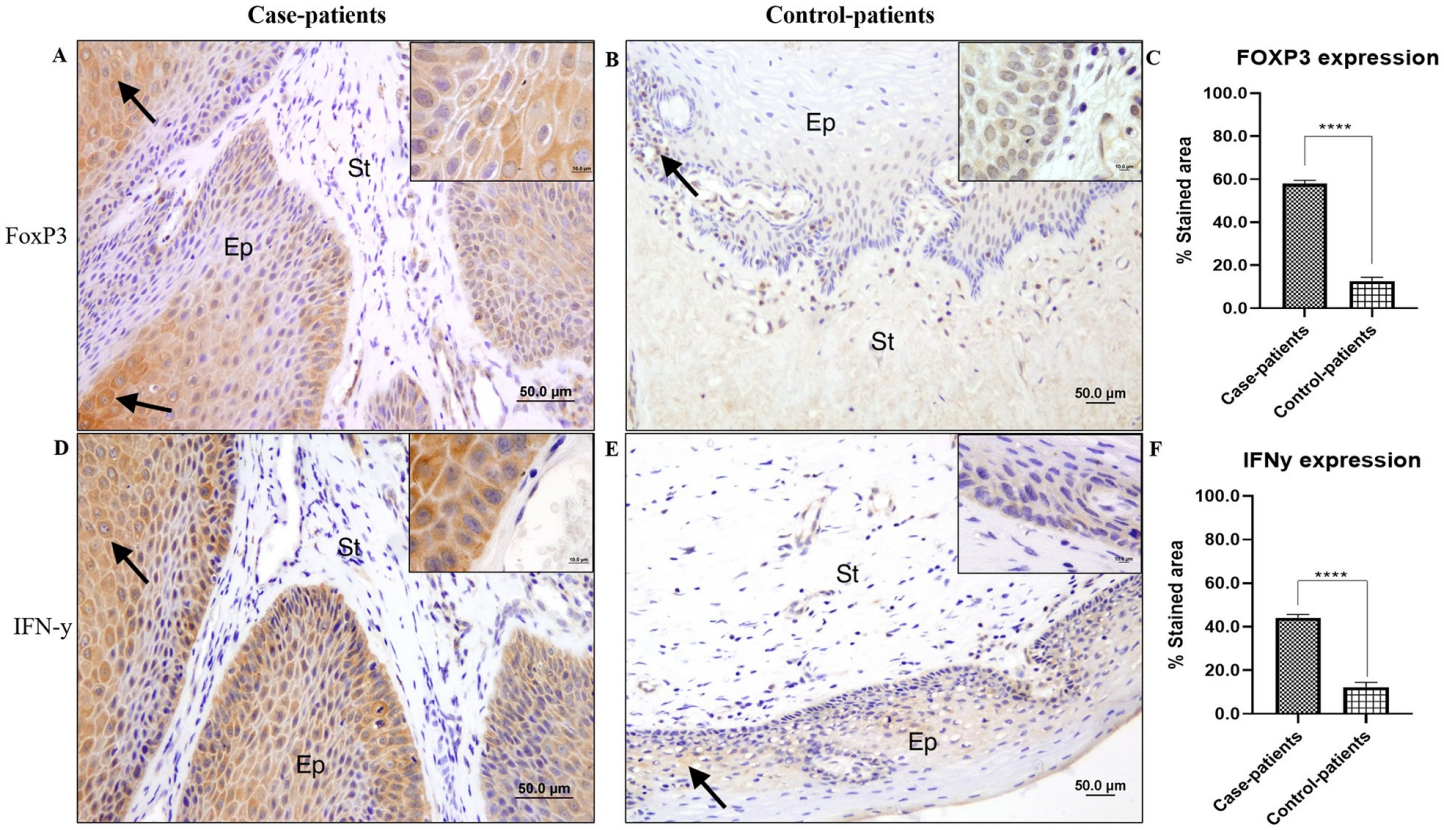
Immunohistochemical assays. Photomicrographs and graphics showing the cytoplasmatic expression pattern of FOXP3 (A-C) and IFN-γ (D-F). Scale bars: 50 μM in the main pictures and 10 μM in the insets. ANOVA was followed by the Tukey`s Multiple comparison test (± S.E.M. **** = p < 0.0001). Arrows: positive immunostaining for different markers; Ep: stratified squamous epithelium; St: connective tissue stroma of the dermis.

**Fig 4:**
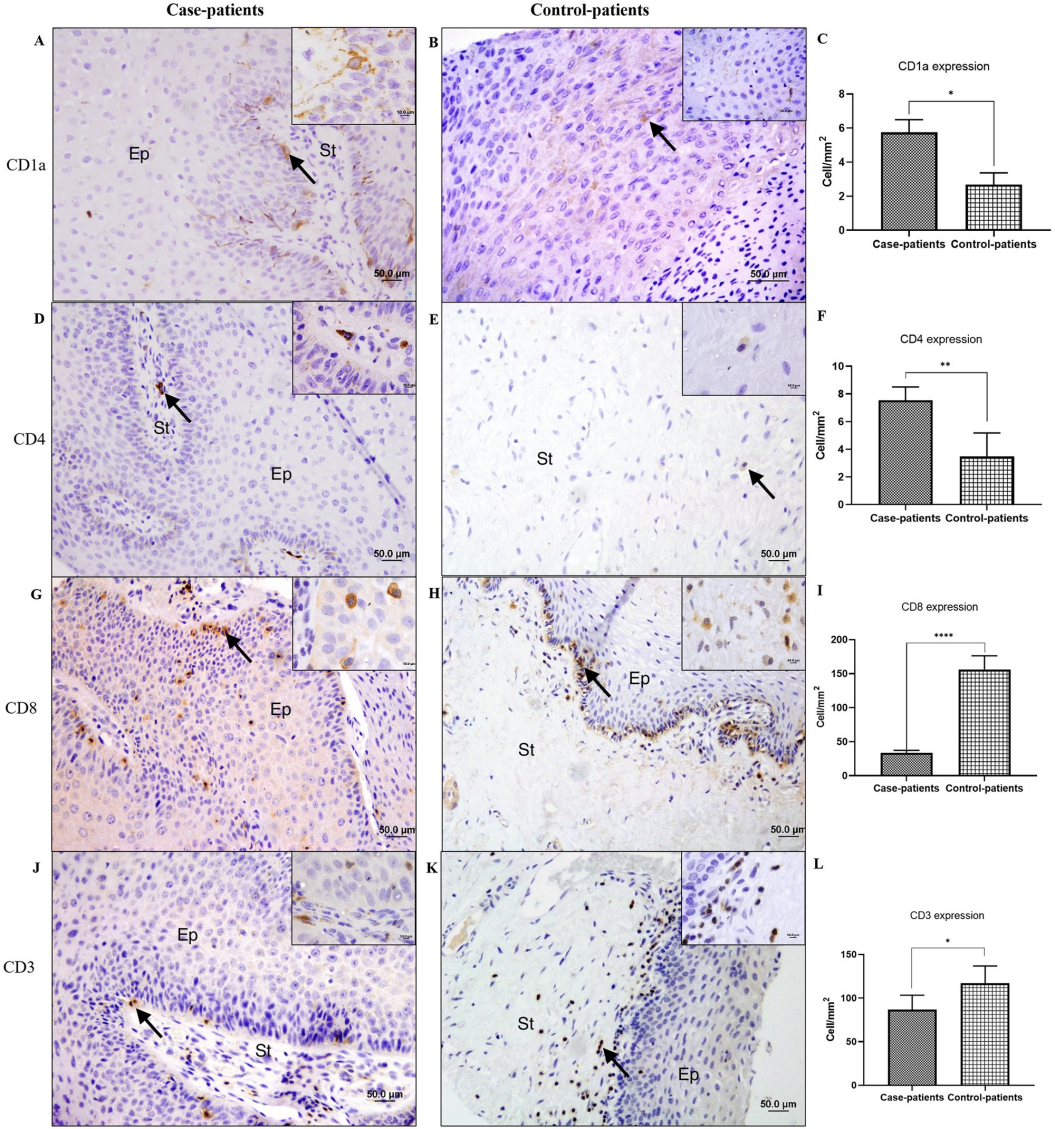
Immunohistochemical assays. Photomicrographs and graphics showing the punctual staining pattern of CD1a, corresponding to Langerhans cells (A-C), CD4 (D-F), CD8 (G-I), and CD3 (J-L) expression. Scale bars: 50 μM in the main pictures and 10 μM in the insets. ANOVA was followed by the Tukey`s Multiple comparison test (± S.E.M. * = p < 0.01, ** = p < 0,001 and **** = p < 0.0001). Arrows: positive immunostaining for different markers; Ep: stratified squamous epithelium; St: connective tissue stroma of the dermis.

Six different immunomarkers (CD1a, FOXP3, CD3, CD4, CD8, and IFN-γ) were used to quantify immune cells in CA samples. From the genes validated by RT-qPCR, immunomarker selection was performed following the innate and adaptive immune pathways. For FOXP3 and INF-γ, a cytoplasmic pattern was observed among cells of the stratified squamous epithelium (Fig 3A, 3B, 3D and 3E). For CD1a, as expected for phagocytic Langerhans cells, punctual staining was characterized by thin cytoplasmic extensions among epithelial cells (Fig 4A and 4B); for CD4, CD3, and CD8, cytoplasmic staining was observed but with a well-delimited pattern among stromal cells (CD4 Fig 4D and 4E) or both stromal and epithelial cells (CD8 Fig 4G and 4H, and CD3 Fig 4J and 4K).

FOXP3 expression presented a three-fold increase in the case-patients TMA compared to that from the control-patients TMA (Fig 3A–3C) (p < 0.0001). IFN-y expression was also significantly different (p < 0.0001) between case-patients and control-patients, as shown by the staining index (Fig 3D–3F). There was a 3.5-fold increase in the expression of this cytokine in CA samples compared to that in control tissues.

For the CD1a immunomarker, average counts of 5.74 and 2.68 cells/mm^2^ were obtained in TMAs from case- and control-patients, respectively. CD1a expression was approximately twice as high in case-patient samples compared to that in the control sample group (Fig 4A–4C), showing a significant difference between groups (p = 0.0318). CD1a+ LCs were present in the epithelium, with a prevalence in the basal stratum, showing cytoplasm-specific staining and a dendritic appearance (Fig 4A).

CD4 levels increased by more than two-fold in case samples (mean of 7.53 cells/mm^2^) than in control samples (mean of 3.47 cells/mm^2^) (p = 0.0049) (Fig 4D–4F).

CD8 expression showed a four-fold decrease in case patients (p < 0.0001) (Fig 4G–4I) compared to that in control tissue samples. Levels of CD3 in case samples were also significantly lower than those in control samples (p = 0.0329) (Fig 4J–4L).

## Discussion

HPV types that cause genital warts do not generally cause cancerous changes. Most HPV infections are transient, but in 10% of patients, the infection persists and may produce late recurrence, especially in states of immune deficiency [34].

In this study, 31 immune system genes were found to be differentially expressed in condyloma acuminata (CA) lesions. Some gene expression heterogeneity was also observed among the evaluated samples. This was probably attributed to the fact that human immune systems vary among individuals [35, 36]. Analysis of cells and proteins from the immune system in healthy adults revealed inter-individual variation [37–40]. Overall, heritable and non-heritable factors, such as the differential expression of immune system proteins and pathogen infection, can explain most of the variation [35, 36] and may be responsible for individual cellular responses at the time of sample collection.

As the primary target of HPV, keratinocytes play an important role in the emergence of viral infection and subsequently promoting an effective immune response. As part of the innate response, the immune defense of the epithelium affects the participation of dendritic cells, represented by LCs and keratinocytes [41]. Keratinocytes act as antigen-presenting cells and can induce cytokine expression by expressing Toll-like receptors (TLRs) [42]. Toll-like receptors recognize pathogen-associated molecular patterns (PAMPs) and promote cytokine production, leading to a pro-inflammatory environment [14].

Epidermal Langerhans cells (LCs) are immature skin dendritic cells. Changes in the number of local dendritic cells can result in immune system damage and contribute to viral persistence [43].

To analyze the presence of LCs in condylomas, we used the CD1a antibody. CD1a serves as a surface marker for LCs, as it is expressed by LCs participating in antigen presentation. We found a higher expression of LCs in test samples than in control tissue samples.

In contrast to our results, Pan and co-authors observed fewer LCs in CA lesions compared to those in normal tissues. The same result was observed by Feng and co-authors when investigating the frequency of LCs in CA lesions and normal controls using CD1a staining by immunohistochemistry [41, 43].

One reason for our contrasting results could be the control exerted by miRNAs, as evidenced by McBee and co-authors in 2011; they reported a possible control of the CD1A gene by miR-344 in cervical dysplasia samples. The same mechanism could be present in condyloma acuminatum, as miR-433 involvement in cellular proteins targeted by high-risk or low-risk E6 and E7 HPV proteins has not been examined. Thus, additional studies are required to confirm this hypothesis. Furthermore, differential expression of LCs in condyloma indicates the innate immune activity as an initial control for HPV. Similarly, our gene expression results also showed upregulation of *GZMB*, which is known to activate target cell apoptosis through caspase-dependent perforin [44], a protein expressed by natural killer (NK) cells as part of the innate immune system [45].

IFN-γ is a soluble cytokine belonging to the IFN type II class and is secreted by cells of both the innate and adaptive immune systems [46]. Based on the results obtained here, this gene was overexpressed in the analyzed condyloma acuminata samples. Immunohistochemical analysis also revealed the overexpression of this cytokine in CA samples, as previously described by *IFNG* overexpression in keratinocytes, which play an important role during the triggering of HPV infection and are considered immune sentinels [9, 14]. Hu and co-authors (2015) also reported increased *IFNG* expression in HPV-positive tissues [47].

The involvement of T helper 1 (Th1) cells in host defense against HPV was evidenced in this study by the overexpression of CD4, along with overexpression of *IFNG*, *IL12B*, and *IL8* in genital warts. Strong CD4+ T helper (Th) and cytotoxic T cell (CTL) responses are essential for effective HPV clearance. Th1 mechanisms (IFN-γ, IL-6, TNF-α, IL-12, and IL-8) act against intracellular pathogens and promote pro-inflammatory responses [48]. Furthermore, a higher count of CD4 and FOXP3 immunomarkers in condyloma tissues than in control tissues, as demonstrated herein, suggests activation of the adaptive immune response. FOXP3 is expressed by regulatory T cells (Treg cells), and its upregulation can indicate regulatory activity [49].

Gene expression analysis showed downregulation of *NFATC4*, which encodes a member of the nuclear factor of activated T cell (NFAT) protein family [50, 51]. The encoded protein is involved in the inducible expression of cytokine genes in T cells, particularly Th2 cells [52]. As we observed activation of T cells in CA, represented by the upregulation of CD4+ and FOXP3, we expected to find upregulated *NFATC4* expression. The opposite result could be a consequence of *IL-7* downregulation, which was also observed in the present study. IL-7 is reported to mediate the NFAT activation pathway independent of Ca^2+^/calcineurin signaling [53], an NFAT canonical activation signaling pathway critical for early thymocyte development [54].

Contrary to the higher number of CD4+ cells in CA samples, immunohistochemical analysis revealed fewer CD8+ cells in CA compared to those in the control tissues. Class I MHC complexes restricted to CD8+ T cells are a principal component of adaptive immune responses to intracellular pathogens and tumors [55]. During acute immune responses to viruses or tumor antigens, naive CD8+ T cells undergo rapid proliferation and differentiation after antigen recognition [55].

A powerful CD8**+** T-cell response is crucial for host defense against HPV persistence and suppression of HPV-associated disease progression [56–58].

The levels of CD8^**+**^ T-cell infiltration positively correlate with the regression of HPV-positive lesions [59]. In 2017, Bhat et al. demonstrated that the downregulation of CD8**+** T-cell cytotoxicity in HPV-related lesions may be caused by suppression via E7-expressing keratinocytes [60]. Papillomaviruses remain in the lowest epithelial layers and persist as episomes in these cells. Under these conditions, HPV genome replication occurs according to the differentiating/proliferating basal cells with limited viral gene expression to reduce the host immune response. Thus, viral proteins such as E7 and E1 may be dispensable for maintaining replication [61] with E2 being one of the limited number of essential viral gene products required for genome maintenance [62]. Therefore, from the results obtained here, the low expression of CD8+ T-cells could be related to the persistence of lesions caused by HPV, as CD8+ T-cells can induce death of infected cells.

CD3 staining represents the T cells in general [63], and its high levels in control tissues may be related to the elevated expression of CD8+ T-cells observed in the same sample group.

In summary, the results presented here show differentially expressed genes (DEGs) selected after the initial screening of 92 immune-related genes in CA samples. To confirm these results, six genes were validated: *GZMB*, *IFNG*, *NFATC4*, *IL7*, *IL8* and *IL12B*. Of these, *GZMB*, *IFNG*, *IL8*, and *IL12B* were upregulated, whereas *NFATC4* and *IL7* were downregulated in the analyzed CA samples. Thus, we observed an increase in the expression levels of pro-inflammatory cytokines and genes involved in the innate immune response and activation of adaptive immunity. The increased expression levels were reflected in the protein levels of the CA samples, as evidenced by the immunomarkers FOXP3, IFN-γ, CD1a, and CD4. These results suggested that the initial host defense against HPV was accompanied at some level by the activation of T lymphocytes (high expression of FOXP3 and CD4 immunomarkers). Downregulation of CD3 and CD8 expression was also observed.

In conclusion, increased levels of pro-inflammatory cytokines in HPV-positive patients compared with those in HPV-negative patients seem to reflect the elevated immunogenicity of HPV-positive CA lesions. Host defense against HPV begins during the early stages of the innate immune response and is followed by the activation of T lymphocytes, which are mainly represented by CD4+ and regulatory T cells. The low CD8+ T cell count in CA may contribute to this recurrent behavior. Additional studies may contribute to further elucidate the mechanism of host defense against HPV infection in condyloma acuminata.

## Supporting information

S1 Fig Heatmap of 92 differentially expressed genes in CA samples. A numerical version is presented in S5 Table. Highlight for the 31 DEGs.
(TIF)

S2 Fig DNA sequence of NESTED-PCR product downstream of GP5+ oligonucleotide for genotyping. **(A)** BLAST alignment analysis evidence HPV genotype 6 based on the database stored in the GenBank and **(B)** BLAST alignment analysis evidence HPV genotype 11 based on the database stored in the GenBank. The GP5+ oligonucleotide binding site is highlighted.
(TIF)

S3 Fig Illustrative diagrams. (A) diagram illustrating the experimental plan of the study. (B) diagram summarizing the findings of the study.
(TIF)

S1 Table Immune genes contained in the TaqMan array human immune, fast 96-well plate. (DOCX)

S2 Table TaqMan gene expression assays identification. (DOCX)

S3 Table Markers, specific targets, and dilution applied. (DOCX)

S4 Table Occurrence of HPV 6 and HPV11 in fresh condyloma acuminata samples. (DOCX)

S5 Table 92 immune-related genes differentially expressed in condyloma acuminata biopsies compared to controls. Relative mRNA expression level (Log_2_). *GAPDH*, *18S*, *GUSB* and *HPRT1* are reference genes.
(DOCX)

S6 Table Immune-related genes differentially expressed in condyloma acuminata biopsies compared to controls. Relative mRNA expression level (Log_2_).
(DOCX)

S7 Table Patient´s characteristics of 27 fresh condyloma acuminata biopsies and normal tissues. (DOCX)

S1 File (DOCX)
