# Supplementary figures and images for: Condyloma acuminata: An evaluation of the immune response at cellular and molecular levels
Source: PLoS One. 2023 Apr 13;18(4):e0284296. doi: 10.1371/journal.pone.0284296 (PMC10101375; doi:10.1371/journal.pone.0284296)

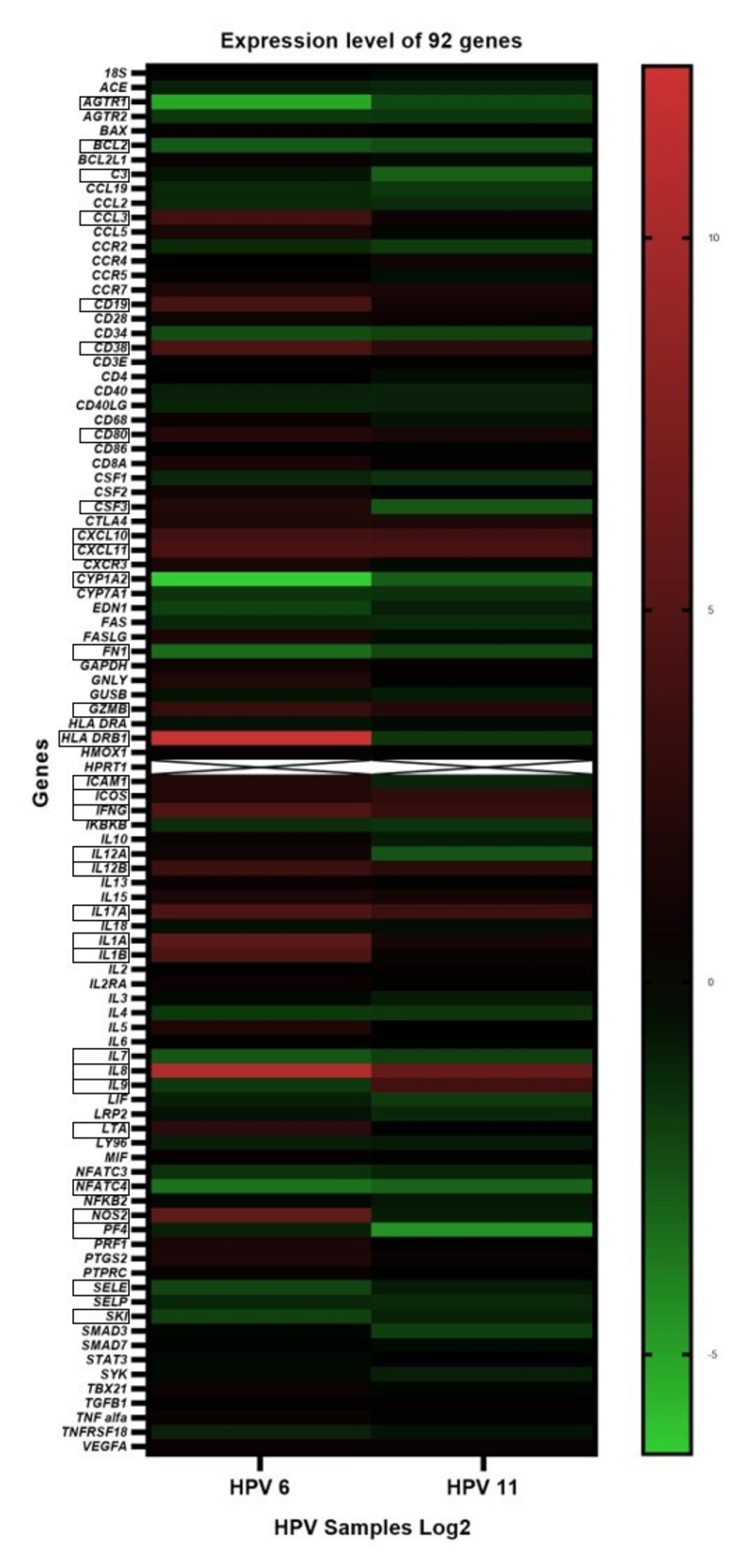

Supplement: S1 Fig — A numerical version is presented in S5 Table. Highlight for the 31 DEGs. (TIF) [file pone.0284296.s001.tif]

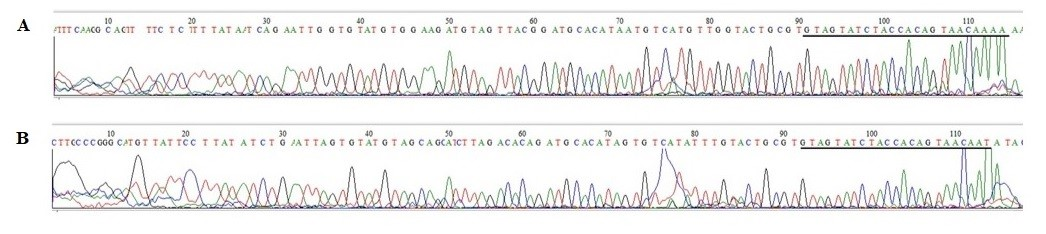

Supplement: S2 Fig — (A) BLAST alignment analysis evidence HPV genotype 6 based on the database stored in the GenBank and (B) BLAST alignment analysis evidence HPV genotype 11 based on the database stored in the GenBank. The GP5+ oligonucleotide binding site is highlighted. (TIF) [file pone.0284296.s002.tif]

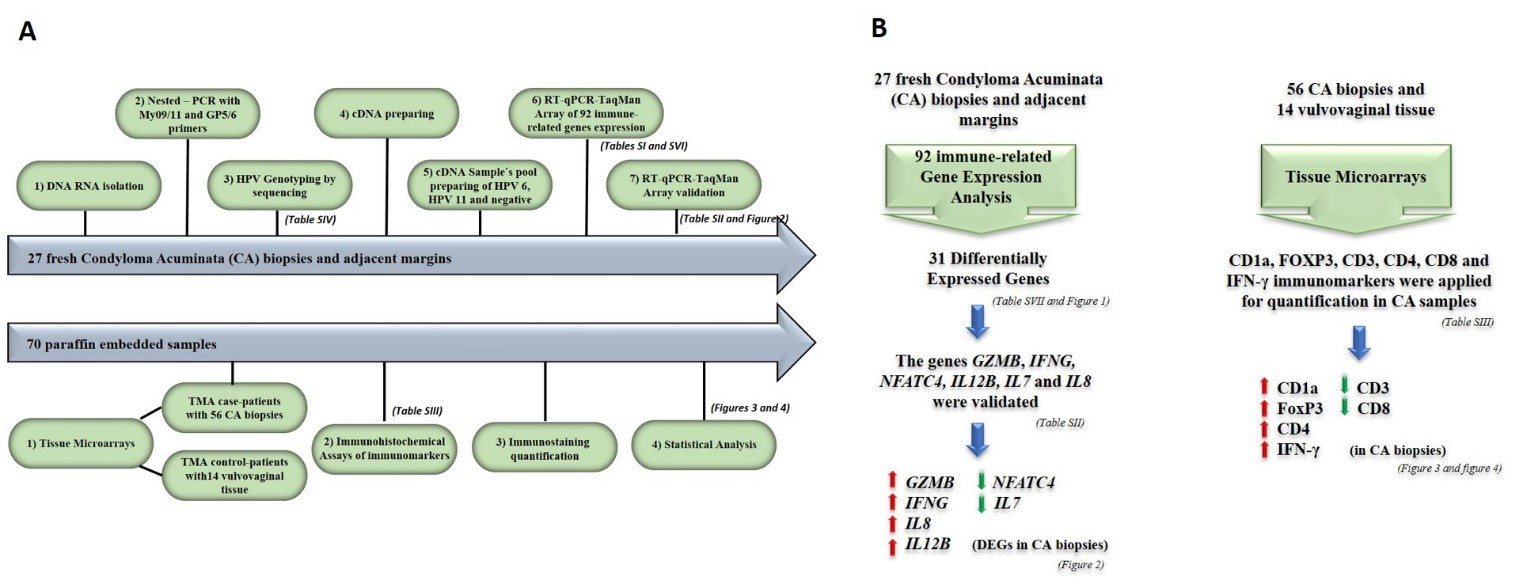

Supplement: S3 Fig — (A) diagram illustrating the experimental plan of the study. (B) diagram summarizing the findings of the study. (TIF) [file pone.0284296.s003.tif]
