# Supplementary material for: Condyloma acuminata: An evaluation of the immune response at cellular and molecular levels
Source: PLoS One. 2023 Apr 13;18(4):e0284296. doi: 10.1371/journal.pone.0284296 (PMC10101375; doi:10.1371/journal.pone.0284296)
Supplement: S1 Table — (DOCX) [file pone.0284296.s004.docx]

| **Assay ID** | **Gene Symbol** |
| --- | --- |
| Hs99999905_m1 | *GAPDH* |
| Hs99999909_m1 | *HPRT1* |
| Hs99999908_m1 | *GUSB* |
| Hs00174092_m1 | *IL1A* |
| Hs00174097_m1 | *IL1B* |
| Hs00174114_m1 | *IL2* |
| Hs00174117_m1 | *IL3* |
| Hs00174122_m1 | *IL4* |
| Hs00174200_m1 | *IL5* |
| Hs00174131_m1 | *IL6* |
| Hs00174202_m1 | *IL7* |
| Hs00174103_m1 | *IL8* |
| Hs00174125_m1 | *IL9* |
| Hs00174086_m1 | *IL10* |
| Hs00168405_m1 | *IL12A* |
| Hs00233688_m1 | *IL12B* |
| Hs00174379_m1 | *IL13* |
| Hs00174106_m1 | *IL15* |
| Hs00174383_m1 | *IL17A* |
| Hs00155517_m1 | *IL18* |
| Hs00234142_m1 | *CCL3* |
| Hs00171149_m1 | *CCL19* |
| Hs00234140_m1 | *CCL2* |
| Hs00174575_m1 | *CCL5* |
| Hs00174150_m1 | *CCR2* |
| Hs99999919_m1 | *CCR4* |
| Hs00152917_m1 | *CCR5* |
| Hs00171054_m1 | *CCR7* |
| Hs00171041_m1 | *CXCR3* |
| Hs00171042_m1 | *CXCL10* |
| Hs00171138_m1 | *CXCL11* |
| Hs00174164_m1 | *CSF1* |
| Hs00171266_m1 | *CSF2* |
| Hs00357085_g1 | *CSF3* |
| Hs00234174_m1 | *STAT3* |
| Hs00174517_m1 | *NFKB2* |
| Hs00233284_m1 | *IKBKB* |
| Hs00167894_m1 | *CD3E* |
| Hs00181217_m1 | *CD4* |
| Hs00233520_m1 | *CD8A* |
| Hs00174333_m1 | *CD19* |
| Hs00166229_m1 | *IL2RA* |
| Hs00174796_m1 | *CD28* |
| Hs00233552_m1 | *CD38* |
| Hs00374176_m1 | *CD40* |
| Hs00365634_g1 | *PTPRC* |
| Hs00154355_m1 | *CD68* |
| Hs00175478_m1 | *CD80* |
| Hs00199349_m1 | *CD86* |
| Hs00175480_m1 | *CTLA4* |
| Hs00163934_m1 | *CD40LG* |
| Hs00219575_m1 | *HLA DRA* |
| Hs99999917_m1 | *HLA DRB1* |
| Hs00203436_m1 | *TBX21* |
| Hs00188346_m1 | *TNFRSF18* |
| Hs00359999_m1 | *ICOS* |
| Hs00167248_m1 | *NOSII* |
| Hs00153350_m1 | *BCL2* |
| Hs00169141_m1 | *BCL2L1* |
| Hs00180269_m1 | *BAX* |
| Hs00164932_m1 | *ICAM1* |
| Hs00174583_m1 | *SELP* |
| Hs00174057_m1 | *SELE* |
| Hs00157965_m1 | *HMOX1* |
| Hs00153133_m1 | *PTGSII* |
| Hs00189742_m1 | *LRP2* |
| Hs00167927_m1 | *CYP1A2* |
| Hs00167982_m1 | *CYP7A1* |
| Hs00174143_m1 | *IFNG* |
| Hs00169473_m1 | *PRF1* |
| Hs00188051_m1 | *GZMB* |
| Hs00246266_m1 | *GNLY* |
| Hs00163653_m1 | *FAS* |
| Hs00181225_m1 | *FASLG* |
| Hs00171257_m1 | *TGFB1* |
| Hs00232222_m1 | *SMAD3* |
| Hs00178696_m1 | *SMAD7* |
| Hs00161707_m1 | *SKI* |
| Hs00365052_m1 | *FN1* |
| Hs00163811_m1 | *C3* |
| Hs00174128_m1 | *TNF* |
| Hs00236874_m1 | *LTA* |
| Hs00174179_m1 | *ACE* |
| Hs00173626_m1 | *VEGFA* |
| Hs00156373_m1 | *CD34* |
| Hs00241341_m1 | *AGTR1* |
| Hs00169126_m1 | *AGTR2* |
| Hs00174961_m1 | *EDN1* |
| Hs00171455_m1 | *LIF* |
| Hs00209771_m1 | *LY96* |
| Hs00236988_g1 | *MIF* |
| Hs00190046_m1 | *NFATC3* |
| Hs00190037_m1 | *NFATC4* |
| Hs00236998_m1 | *PF4* |
| Hs00374292_m1 | *SYK* |
