# Supplementary material for: Condyloma acuminata: An evaluation of the immune response at cellular and molecular levels
Source: PLoS One. 2023 Apr 13;18(4):e0284296. doi: 10.1371/journal.pone.0284296 (PMC10101375; doi:10.1371/journal.pone.0284296)
Supplement: S2 Table — (DOCX) [file pone.0284296.s005.docx]

| **Gene symbol** | **Assay ID** |
| --- | --- |
| *HPRT1** | Hs99999909_m1 |
| *GZMB* | Hs00188051_m1 |
| *IFNG* | Hs00174143_m1 |
| *NFATC4* | Hs00190037_m1 |
| *IL7* | Hs00174202_m1 |
| *IL12B* | Hs00233688_m1 |
| *IL8* | Hs00174103_m1 |

***Endogenous gene**
