# Supplementary material for: Condyloma acuminata: An evaluation of the immune response at cellular and molecular levels
Source: PLoS One. 2023 Apr 13;18(4):e0284296. doi: 10.1371/journal.pone.0284296 (PMC10101375; doi:10.1371/journal.pone.0284296)
Supplement: S3 Table — (DOCX) [file pone.0284296.s006.docx]

| **Primary antibody** | **Target** | **Dilution** | **Label** |
| --- | --- | --- | --- |
| **anti-FOXP3 (*mouse)*** | T reg cells | 1:100 | ab20034, Abcam |
| **anti-CD1a (*mouse)*** | Langerhans cells | 1:50 | ab708, Abcam |
| **anti-CD4 (*mouse*)** | T helper cells | 1:25 | ab846, Abcam |
| **anti-CD8 (*mouse*)** | Cytotoxic T cells | 1:50 | ab17147, Abcam |
| **anti-CD3 (*rabbit*)** | T Cells | 1:50 | ab16669, Abcam |
| **anti-IFN-γ (*rabbit*)** | Cytokine interferon gamma | 1:500 | ab9657, Abcam |
