# Supplementary material for: Condyloma acuminata: An evaluation of the immune response at cellular and molecular levels
Source: PLoS One. 2023 Apr 13;18(4):e0284296. doi: 10.1371/journal.pone.0284296 (PMC10101375; doi:10.1371/journal.pone.0284296)
Supplement: S4 Table — (DOCX) [file pone.0284296.s007.docx]

| **HPV type** | **Number of Patients** | **Percentage** |
| --- | --- | --- |
| **HPV 6** | 19 | 70,4% |
| **HPV 11** | 8 | 29,6% |
