# Supplementary material for: Condyloma acuminata: An evaluation of the immune response at cellular and molecular levels
Source: PLoS One. 2023 Apr 13;18(4):e0284296. doi: 10.1371/journal.pone.0284296 (PMC10101375; doi:10.1371/journal.pone.0284296)
Supplement: S5 Table — Relative mRNA expression level (Log2). GAPDH, 18S, GUSB and HPRT1 are reference genes. (DOCX) [file pone.0284296.s008.docx]

| **Gene** | **HPV 6 samples (Log_2_)** | **HPV 11 samples (Log_2_)** |
| --- | --- | --- |
| *18S* | -0,1 | -0,255 |
| *ACE* | -1,009 | -1,239 |
| *AGTR1* | -5,186 | -2,231 |
| *AGTR2* | -1,713 | -1,674 |
| *BAX* | 0,343 | 0,016 |
| *BCL2* | -2,715 | -2,368 |
| *BCL2L1* | 0,537 | -0,354 |
| *C3* | -0,661 | -2,939 |
| *CCL19* | -1,256 | -1,702 |
| *CCL2* | -1,255 | -1,381 |
| *CCL3* | 3,877 | 0,81 |
| *CCL5* | 1,528 | -0,215 |
| *CCR2* | -1,323 | -1,843 |
| *CCR4* | 0,05 | 0,945 |
| *CCR5* | 0,402 | -0,46 |
| *CCR7* | 1,693 | 1,48 |
| *CD19* | 4,166 | 1,092 |
| *CD28* | 0,774 | 0,658 |
| *CD34* | -2,407 | -2,072 |
| *CD38* | 4,588 | 2,568 |
| *CD3E* | -0,153 | 0,238 |
| *CD4* | 0 | -0,449 |
| *CD40* | -0,952 | -1,036 |
| *CD40LG* | -1,12 | -1,042 |
| *CD68* | 0,62 | -0,631 |
| *CD80* | 2,184 | 1,451 |
| *CD86* | 0,055 | -0,101 |
| *CD8A* | 1,411 | 0,407 |
| *CSF1* | -1,177 | -1,543 |
| *CSF2* | 1,085 | 0,005 |
| *CSF3* | 1,972 | -2,741 |
| *CTLA4* | 1,748 | 1,733 |
| *CXCL10* | 3,864 | 3,734 |
| *CXCL11* | 4,402 | 4,269 |
| *CXCR3* | 1,456 | -0,344 |
| *CYP1A2* | -6,339 | -2,893 |
| *CYP7A1* | -1,601 | -1,485 |
| *EDN1* | -1,982 | -0,955 |
| *FAS* | -1,247 | -1,352 |
| *FASLG* | 1,561 | -0,396 |
| *FN1* | -3,367 | -2,21 |
| *GAPDH* | 0,982 | 0,376 |
| *GNLY* | 1,928 | -0,118 |
| *GUSB* | -0,568 | -0,851 |
| *GZMB* | 3,123 | 2,093 |
| *HLA DRA* | -0,55 | -0,263 |
| *HLA DRB1* | 12,302 | -1,674 |
| *HMOX1* | 0,379 | 0,117 |
| *HPRT1* | - | - |
| *ICAM1* | 2,029 | -1,089 |
| *ICOS* | 1,988 | 2,718 |
| *IFNG* | 4,844 | 3,272 |
| *IKBKB* | -1,403 | -1,606 |
| *IL10* | 0,595 | -0,847 |
| *IL12A* | 0,978 | -2,586 |
| *IL12B* | 3,495 | 2,52 |
| *IL13* | 0,625 | -0,15 |
| *IL15* | 1,521 | 1,222 |
| *IL17A* | 4,632 | 3,509 |
| *IL18* | -0,506 | -0,413 |
| *IL1A* | 5,484 | 1,358 |
| *IL1B* | 4,656 | 0,496 |
| *IL2* | -0,087 | 0,361 |
| *IL2RA* | 0,626 | 0,22 |
| *IL3* | -0,257 | -0,805 |
| *IL4* | -1,713 | -1,674 |
| *IL5* | 1,893 | 0,025 |
| *IL6* | 0,234 | -0,06 |
| *IL7* | -2,626 | -1,948 |
| *IL8* | 10,643 | 6,181 |
| *IL9* | -1,713 | 3,936 |
| *LIF* | -0,932 | -1,821 |
| *LRP2* | -0,632 | -1,243 |
| *LTA* | 2,566 | 0,055 |
| *LY96* | -0,933 | -0,806 |
| *MIF* | 0,413 | 0,229 |
| *NFATC3* | -1,502 | -1,145 |
| *NFATC4* | -3,555 | -3,094 |
| *NFKB2* | -0,296 | -0,683 |
| *NOS2* | 5,731 | -0,877 |
| *PF4* | -0,991 | -4,584 |
| *PRF1* | 1,593 | 0,038 |
| *PTGS2* | 1,732 | 0,541 |
| *PTPRC* | 0,421 | -0,078 |
| *SELE* | -2,104 | -0,838 |
| *SELP* | -1,189 | -1,274 |
| *SKI* | -2,044 | -1,058 |
| *SMAD3* | -0,158 | -1,921 |
| *SMAD7* | -0,115 | -0,317 |
| *STAT3* | -0,29 | -0,04 |
| *SYK* | -0,266 | -0,939 |
| *TBX21* | 0,695 | -0,196 |
| *TGFB1* | 0,233 | 0,169 |
| *TNF alfa* | 0,526 | -0,074 |
| *TNFRSF18* | -1,04 | -0,596 |
| *VEGFA* | 0,627 | 0,581 |
