# Supplementary material for: Condyloma acuminata: An evaluation of the immune response at cellular and molecular levels
Source: PLoS One. 2023 Apr 13;18(4):e0284296. doi: 10.1371/journal.pone.0284296 (PMC10101375; doi:10.1371/journal.pone.0284296)
Supplement: S6 Table — Relative mRNA expression level (Log2). (DOCX) [file pone.0284296.s009.docx]

| **Gene** | **Description** | **HPV 6 samples (Log_2_)** | **HPV 11 samples (Log_2_)** |
| --- | --- | --- | --- |
| ***AGTR1*** | Angiotensin II receptor type 1 | -5,1 | -2,2 |
| ***BCL2*** | B-cell CLL/lymphoma 2 | -2,7 | -2,4 |
| ***C3*** | Complement C3 | -0,6 | -2,9 |
| ***CCL3*** | Chemokine (C-C motif) ligand 3 | 3,9 | 0,8 |
| ***CD19*** | CD19 molecule | 4,2 | 1,1 |
| ***CD38*** | CD38 molecule | 4,6 | 2,6 |
| ***CD80*** | CD80 molecule | 2,2 | 1,4 |
| ***CSF3*** | Colony stimulating factor 3 | 1,9 | -2,7 |
| ***CXCL10*** | [C-X-C motif chemokine ligand 10](https://www.ncbi.nlm.nih.gov/gene/3627) | 3,8 | 3,7 |
| ***CXCL11*** | C-X-C motif chemokine ligand 11 | 4,4 | 4,3 |
| ***CYP1A2*** | [Cytochrome P450 family 1 subfamily A member 2](https://www.ncbi.nlm.nih.gov/gene/1544) | -6,3 | -2,8 |
| ***FN1*** | [Fibronectin 1](https://www.ncbi.nlm.nih.gov/gene/2335) | -3,3 | -2,2 |
| ***GZMB*** | [Granzyme B](https://www.ncbi.nlm.nih.gov/gene/3002) | 3,1 | 2,1 |
| ***HLADRB1*** | M[ajor Histocompatibility Complex, class II, DR beta 1](https://www.ncbi.nlm.nih.gov/gene/3123) | 12 | -1,7 |
| ***ICAM1*** | [Intercellular adhesion molecule 1](https://www.ncbi.nlm.nih.gov/gene/3383) | 2 | -1,1 |
| ***ICOS*** | [Inducible T-cell costimulator](https://www.ncbi.nlm.nih.gov/gene/29851) | 1,98 | 2,7 |
| ***IFNG*** | [Interferon gamma](https://www.ncbi.nlm.nih.gov/gene/3458) | 4,8 | 3,3 |
| ***IL12A*** | Interleukin 12B | 0,98 | -2,6 |
| ***IL12B*** | Interleukin 12B | 3,5 | 2,5 |
| ***IL17A*** | Interleukin 17A | 4,6 | 3,5 |
| ***IL1A*** | Interleukin 1A | 5,5 | 1,4 |
| ***IL1B*** | Interleukin 1B | 4,6 | 0,5 |
| ***IL7*** | Interleukin 7 | -2,6 | -1,99 |
| ***IL8*** | Interleukin 8 | 10,6 | 6,2 |
| ***IL9*** | Interleukin 9 | -1,7 | 3,9 |
| ***LTA*** | [Lymphotoxin alpha](https://www.ncbi.nlm.nih.gov/gene/4049) | 2,6 | 0 |
| ***NFATC4*** | [Nuclear factor of activated T-cells 4](https://www.ncbi.nlm.nih.gov/gene/4776) | -3,55 | -3,1 |
| ***NOSII*** | Nitric oxide synthase 2, inducible | 5,7 | -0,9 |
| ***PF4*** | [Platelet factor 4](https://www.ncbi.nlm.nih.gov/gene/5196) | -0,99 | -4,6 |
| ***SELE*** | [Selectin E](https://www.ncbi.nlm.nih.gov/gene/6401) | -2,1 | -0,84 |
| ***SKI*** | SKI proto-oncogene | -2 | -1 |
