# Supplementary material for: Condyloma acuminata: An evaluation of the immune response at cellular and molecular levels
Source: PLoS One. 2023 Apr 13;18(4):e0284296. doi: 10.1371/journal.pone.0284296 (PMC10101375; doi:10.1371/journal.pone.0284296)
Supplement: S7 Table — (DOCX) [file pone.0284296.s010.docx]

| **Patient´s Characteristics** | **Number (%)** | ***p-value*** |
| --- | --- | --- |
| ***Age (Years)*** |  | 0,2311 |
| 22-42 | 19 (70,4) |  |
| 43-63 | 7 (25,9) |  |
| 64-84 | 1 (3,7) |  |
| ***Race*** |  | 0,3228 |
| White | 21 (77,8) |  |
| Black | 6 (22,2) |  |
| ***Marital status*** |  | 0,1750 |
| Single | 17 (63,0) |  |
| Married/cohabiting | 8 (29,6) |  |
| Divorced/separated/widowed | 2 (7,4) |  |
| ***Education*** |  | 0.0935 |
| Primary Education | 12 (44,4) |  |
| Secondary Education | 11 (40,7) |  |
| Graduated | 3 (11,1) |  |
| Unlettered | 1 (3,7) |  |
| ***Smoking status*** |  | 0,3145 |
| Yes | 6 (23,1) |  |
| No | 20 (76,9) |  |
| ***Alcohol abuse*** |  | 0,4471 |
| Yes | 2 (7,7) |  |
| No | 24 (92,3) |  |
