# Supplementary material for: Condyloma acuminata: An evaluation of the immune response at cellular and molecular levels
Source: PLoS One. 2023 Apr 13;18(4):e0284296. doi: 10.1371/journal.pone.0284296 (PMC10101375; doi:10.1371/journal.pone.0284296)
Supplement: S1 File — (DOCX) [file pone.0284296.s011.docx]

## SUPPLEMENTARY MATERIAL

**Total DNA and RNA isolation**

Briefly, 1.0 mL of TRIzol™ (Invitrogen, Carlsbad, California, USA, Catalog number: 15596026) was added to 50–100 mg of tissue. The mixture was homogenized in a sonicator for 15 seconds and then incubated for 5 minutes at room temperature. Next, 200µl of chloroform were added, followed by vortexing for 15 seconds, incubation at room temperature for 3 minutes, and centrifugation at 4°C for 15 minutes at 12,000 × g. The aqueous phase containing RNA was transferred to a new tube and the organic phase was stored at 4°C. For RNA precipitation, 500µl of isopropanol were added and the microtubes were incubated for 10 minutes at room temperature and centrifuged at 4°C for 10 minutes at 12,000 × g. The pellet was washed with 1.0 mL of 75% ethanol and the tubes were centrifuged at 4°C for 5 minutes at 7500 × g. For RNA elution, 50µL of nuclease free water (ThermoFisher Scientific, Waltham, MA, USA, Catalog number: R0601) were added to the pellet. The material was stored at -80°C. For DNA extraction, 300µL of 100% ethanol were added to the organic phase resulting from the RNA extraction. The microtubes were incubated for 3 minutes at room temperature and centrifuged at 4°C for 5 minutes at 2000 × g. The pellet was washed with 1mL of 0.1M sodium citrate in 10% ethanol. After 30 minutes of incubation, the microtubes were centrifuged at 4°C for 5 minutes at 2000 × g and the pellet was resuspended with 1.5 mL of 75% ethanol, followed by incubation for 10 minutes and centrifugation at 4°C for 5 minutes at 2000 × g. For DNA elution, 200µL of nuclease free water were added to the pellet and the material was left overnight at 37ºC. The extracted DNA was stored at -20°C.

The DNA integrity was evaluated via 𝛽-globin endogenous gene amplification by polymerase chain reaction (PCR), generating a 315-bp amplicon ([1](#_ENREF_1)). The RNA and DNA purity (A260/280 nm) were assessed using NanoDrop 2000/2000c (ThermoFisher Scientific, Waltham, MA, USA Catalog number: ND-2000C).

**References**

1. Bernard HU, Chan SY, Manos MM*, et al*: Identification and assessment of known and novel human papillomaviruses by polymerase chain reaction amplification, restriction fragment length polymorphisms, nucleotide sequence, and phylogenetic algorithms. The Journal of infectious diseases 170: 1077-1085, 1994.
